# Assessment of the abdominal wall function after pedicled TRAM flap surgery for breast reconstruction: Use of modified mesh repair for the donor defect

**DOI:** 10.4103/0970-0358.73430

**Published:** 2010

**Authors:** Chacko Cyriac, Ramesh Kumar Sharma, Gurpreet Singh

**Affiliations:** Department of Plastic Surgery, Postgraduate Institute of Medical Education and Research, Chandigarh, India; 1 Department of General Surgery, Postgraduate Institute of Medical Education and Research, Chandigarh, India

**Keywords:** Abdominal wall function, patient questionnaire, pedicled TRAM flap breast reconstruction, straight and lateral curl ups

## Abstract

**Background:**

The pedicled TRAM flap has been a workhorse of autologous breast reconstruction for decades. However, there has been a rising concern about the abdominal wall donor site morbidity with the use of conventional TRAM flap. This has generally been cited as one of the main reasons for resorting to “abdominal wall friendly” techniques. This study has been undertaken to assess the abdominal wall function in patients with pedicled TRAM flap breast reconstruction. The entire width of the muscle and the overlying wide disk of anterior rectus sheath were harvested with the TRAM flap in all our patients and the anterior rectus sheath defect was repaired by a Proline mesh.

**Materials and Methods:**

Abdominal wall function was studied in 21 patients who underwent simultaneous primary unipedicled TRAM flap reconstruction after mastectomy for cancer. In all the patients, the abdominal wall defect was repaired using wide sheet of Proline mesh both as inlay and onlay. The assessment tools included straight and rotational curl ups and a subjective questionnaire. The abdominal wall was also examined for any asymmetry, bulge, or hernia. The minimal follow-up was 6 months postoperative. The objective results were compared with normal unoperated volunteers.

**Results and Conclusions:**

The harvesting the TRAM flap certainly results in changes to the anterior abdominal wall that can express themselves to a variable degree. A relatively high incidence of asymptomatic asymmetry of the abdomen was seen. There was total absence of hernia in our series even after a mean follow-up period of 15.5 months. A few patients were only able to partially initiate the sit up movement and suffered an important loss of strength. In most patients, synergists took over the functional movement but as the load increased, flexion and rotation performances decreased. The lack of correlation between exercise tests and the results of the questionnaire suggests that this statistically significant impairment was functionally not important. The patients encountered little or no difficulty in theis day-to-day activities. Our modification of use of a wide mesh as inlay and onlay repair minimizes the donor site morbidity. This also avoids maneuvers meant for primary closure of the rectus sheath defects, which can result in distortion of umbilicus. Therefore, in conclusion, the unipedicled TRAM flap should be regarded as a valuable option in breast reconstruction provided careful repair of the abdominal wall defect is undertaken using Proline mesh.

## INTRODUCTION

The pedicled TRAM flap has been the workhorse of autologous breast reconstruction since it was first described in 1982.[1] But over the years, many studies have cited the abdominal wall morbidity as a significant complication of this procedure and have begun to favor muscle-sparing procedures such as the DIEP flap as alternatives. These procedures would logically reduce the donor site morbidity, but they are more expensive and technically complex alternatives. In our study, we have followed up 21 pedicled TRAM flap patients to assess the extent of the donor morbidity associated with this procedure and to determine if there really exists a reason for preferring more complex procedures over this simple technique.

## MATERIALS AND METHODS

The study sample included 21 patients who underwent breast reconstruction with the TRAM flap at the PGIMER, Chandigarh, with follow-up time of at least 6 months. Patients with tumor recurrence or distant metastasis were excluded. The control group was represented by female attendants accompanying the patients. The charts were reviewed for any preoperative weakness of the abdomen, details of the surgery, and the incidence of postoperative complications. The patients were clinically evaluated by a physician other than the operating surgeons. The abdomen was examined to detect the existence of any localized tenderness, abnormality in the position of the umbilicus, abdominal wall asymmetry, bulge, or hernia. The definitions adopted by Reece and Kroll[2] were followed in our assessment [Table 1]. The assessment of the abdominal wall muscle function was carried out according to the evaluation of Lacote and Chevalier.[3] The patients and the control subjects were asked to perform straight and rotational curl-ups for assessment of upper and lower rectus and the external oblique muscles on both sides. The patients were given a questionnaire for their opinion about the abdominal wall strength, change of posture, back pain, changes in activities of daily living, and satisfaction with the results of the surgery. Statistical analysis was done using the *t*-test (paired samples test and the independent samples test) and the χ^2^ -test.

**Table 1:** Definition of terms

*Term*	*Definition*
Asymmetry	Unilateral distention of the abdomen, more pronounced when upright, progresses during the day
Bulge	Protrusion of the abdominal wall with palpable margins, no defect in the abdominal wall. No contents to reduce.
Hernia	Protrusion of the abdominal wall with palpable margins with defect involving all musculoaponeurotic layers of abdomen. Contents reducible.

### Operative technique

**Figure 1:**
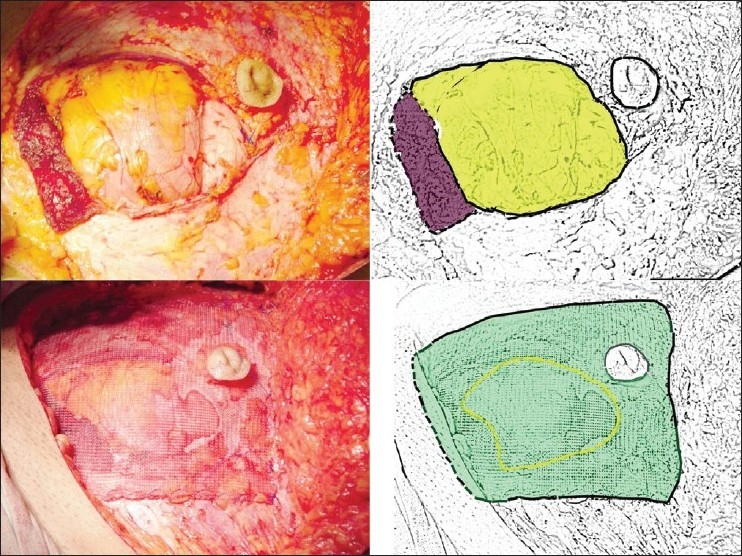
Upper row: defect in the abdominal wall; the lower cut of rectus is depicted in brown and the defect in the wall in yellow in the drawing on left. Lower row: the use of Proline mesh. The mesh spans the defect and is sutured to the edge of defect, and contralateral anterior rectus sheath. The edge of the defect is depicted by broken yellow line and the mesh by green color in the drawing on left

Skin sparing mastectomy with axillary dissection was performed in 15 patients and a total mastectomy with axillary clearance was performed in 6 patients. The reconstructive surgeon harvested an ipsilateral pedicled TRAM flap in all the cases, from the right side in 6 patients and from the left side in 15 patients. Entire width of the rectus muscle with overlying wide disk of anterior rectus sheath was harvested along with the skin island. A Proline mesh was used for repair of the abdominal wall defect in all the patients. The mesh covered the defect and spanned on to the normal contralateral side. The mesh was sutured to the edges of the defect and intact contralateral anterior rectus sheath and the semi-lunar line. The umbilicus was brought out through this mesh [Figure 1].

## RESULTS

The total duration of surgery ranged from 2 h 45 min to 3 h 15 min (mean 3 h). Complications included abdominal seroma (9.5%), minor delay in the healing of the edges of the abdominal flap (9.5%), necrosis of the flap margins (5%), and partial flap necrosis (5%). The average hospital stay was 9.4 days.

The mean follow-up period was 15.5 months (range, 8–32 months). The patients mean age was 46.24 years (range, 20-60 years). The mean postoperative weight was 61.91 kg (range, 53–86 kg) at the time of assessment. The mean BMI calculated was 26.22 kg/m^2^ (range, 22.35–32.37 kg/m^2^). The mean age and BMI among the control group were 43.43 years (range, 25–61 years) and 25.82 kg/m^2^ (range, 22.04–31.56 kg/m^2^), respectively. The *t*-test confirmed both the groups to be well matched and comparable. None of the patients had any evidence of a preoperative deficit in the abdominal wall function.

Table 2 summarizes the results of the clinical evaluation of the patients. Of the patients, 29% had a vague discomfort in the donor site. The χ^2^ -test revealed that this was not significant (*P* = 0.05). No patient had umbilical malpositioning, but 71% had “asymmetry” of the abdominal wall. While 5% had a “bulge” in the donor site, no patient had a true hernia.

**Table 2:** Results of clinical evaluation of patients

*Clinical examination*	*Incidence among total n = 21*
Complaints pertaining to donor site	6 (29%), *P* = 0.05
Umbilical asymmetry	Nil
Abdominal asymmetry	15 (71%), *P* =0.016
Bulge	1 (5%)
Hernia	Nil

There was a distinct difference in straight curl-up performance in the two groups [Table 3]. Of the controls, 95% were able to attain a straight curl-up score of four or five, but none of the TRAM flap patients could achieve the same, and 86% TRAM patients achieved a maximum score of three. The difference between the two groups was found to be highly significant on *t*-test (*P* < 0.01). However, the function of the lower rectus was not significantly impaired in TRAM patients. Seventy-six percent of the controls were able to attain a straight curl-up score of five and 24% a score of four with a mean score of 4.76. Similar results were obtained among the TRAM flap patients (mean score of 4.67). The *t*-test showed that there was no significant difference (*P* = 0.55). The mean upper rectus score was 2.86 while that for the lower rectus was 4.67 in TRAM flap patients. The *t*-test analysis showed that this difference was significant (*P* < 0.01).

**Table 3:** Straight curl ups: Assessment of rectus muscle function

*Score*	*TRAM flap patients (n = 21)*	*Control (n = 21)*
2	3 (14)	–
3	18 (86)	1 (5)
4	Nil	14 (67)
5	Nil	6 (28)

Straight curl ups: upper rectus difference in performance (*P* < 0.01).		

***Score***	***TRAM flap patients (n = 21)***	***Control (n = 21)***

2	–	–
3	1 (5)	–
4	5 (24)	5 (24)
5	15 (71)	16 (76)

Straight curl ups: lower rectus difference in performance (*P* = 0.55).		

***Score***	***Upper rectus (n = 21)***	***Lower rectus (n = 21)***

2	3 (14)	–
3	18 (86)	1 (5)
4	Nil	5 (24)
5	Nil	15 (71)

Straight curl ups: upper rectus vs. lower rectus – difference in performance (*P* < 0.01). Figures in parenthesis are in percentage

Results of the rotational curl-up test [Table 4] were similar to those of the straight curl-up test for the upper rectus. Most TRAM flap patients were able to achieve a score of only two or three (mean score of 2.95 for the right external oblique and 2.52 for the left external oblique). Fifty-seven percent of the controls on the other hand had a score of four, while 9% achieved a score of five (mean score of 3.86). *T*-test showed that this difference was significant (*P* < 0.01). The function of the external oblique on the ipsilateral side showed a mean score of 2.476 and a slightly higher score of 3.00 for the contralateral side. The *T*-test analysis again showed that this difference was significant (*P* < 0.01).

**Table 4:** Rotational curl up: Assessment of oblique muscles

*Score*	*Patients*		*Control*	
	*Right curl up*	*Left curl up*	*Right curl up*	*Left curl up*
2	3	10	–	–
3	16	11	6	6
4	2	Nil	12	12
5	Nil	Nil	3	3

Rotational curl up – difference in performance (*P* < 0.01).				

***Score***		***Ipsilateral***		***Contralateral***

2		11		2
3		10		17
4		Nil		2
5		Nil		Nil

Ipsilateral vs. contralateral external oblique function in TRAM flap patients – difference in performance (*P* < 0.01).

Table 5 shows the results of questionnaire. 10% of our patients felt that there was a decreased abdominal power manifested by a reduced ability to lift heavy objects. Rest of 90% patients did not feel any decease in abdominal strength (*P* = 0.005). None of the patients had any change in the posture, or any increased incidence of back pain following surgery. Moreover, 67% had absolutely no difficulty in getting up from a supine position and 86% required no modifications in their daily activities, which was highly significant on the χ^2^ -test (*P* < 0.01). While 91% were satisfied with the outcome of surgery, 86% were certain that they would recommend this procedure to another patient who would require it.

**Table 5:** Results of the questionnaire

*Question*	*Score*				
	*1*	*2*	*3*	*4*	*5*
Strength of abdomen after surgery	–	4	17	–	–
Change in posture	–	–	21	–	–
Back pain	–	–	21	–	–
Sit up from a lying down posture without help	–	1	2	4	14
Activities at home/place of work	–	–	1	2	18
Recommend procedure to others	–	1	2	11	7
Pleased with outcome	–	1	1	14	5

There was a significant difference between the results of the objective physical evaluation and the results of the subjective assessment (questionnaire) (*P* < 0.01). When the results of the objective assessment were compared to the relatively low incidence of complaints pertaining to the donor site, a statistically significant difference was detected (*P* < 0.05).

## DISCUSSION

The TRAM flap has been the gold standard in autologous breast reconstruction.[4–7] Although it produced superior aesthetic results at the donor site when compared with other options,[8–10] the sacrifice of part of the rectus abdominis muscle and the resulting donor site morbidity has been a major concern.[11–15] We have attempted to study any functional abdominal wall musculature deficit. The role of the rectus abdominis muscle is often overestimated in flexion of the trunk. The gravity is mostly responsible for the flexion of the upper body in most of the daily activities. The rectus muscles are responsible for initiating the movement for the first 30° of flexion, and rest of the movement is completed by the iliopsoas.[16] The rectus sheath enclosing recti is the site of insertion of the other muscles of the anterior abdominal wall. Any deficit involving the rectus muscles or the rectus sheath might, therefore, result in an impairment of the function of the abdominal wall muscles as well.[16]

Only 29% of our patients had any abdominal complaints, mostly a vague discomfort. Asymmetry in the umbilical position was also not observed in our patients. Blondeel *et al*. noted this asymmetry in significant number of his patients.[16] The use of a wide synthetic mesh allows us to maintain umbilical symmetry. Seventy-six percent of our patients had asymmetry of the abdominal wall on the side of harvest of the TRAM flap that was made worse with maneuvers that increased the intra-abdominal pressure. There was no palpable margin for this protrusion. Twenty-four had vague complaints of “slight discomfort” relating to this problem. Rests of the patients were unaware and asymptomatic and the asymmetry was detected on clinical examination by the physician. In 5% patient, a slight bulge was noted, but there was no true hernia. Long-term studies will be necessary to determine if the incidence of hernias will increase in these patients.

A “curl-up” activity involves flexion of the hip joint by the iliopsoas muscles while the rectus muscles have only an isometric stabilizing function.[16] During a “straight curl-up”, the concentric isotonic or dynamic contraction of the rectus muscles and the vertical fibers of the oblique muscles lift the cervical spine followed by the thoracic spine from the surface up to 30°–45°. The iliopsoas muscles take over now and the isometric, static, or stabilizing function of the rectus muscles slowly increase. If the rectus muscles were not active and the trunk could not be stabilized, no sit-up or curl-up could be performed because the iliopsoas muscles would only tilt the pelvis and the lower lumbar spine, leaving the upper body immobile. The full “curl-up”, therefore, clinically evaluates the rectus muscles as flexors at first and as stabilizers later. Following TRAM flap surgery, there is complete loss of function of one of the rectus abdominis muscles. This should lead to a reduction of the strength of the central muscular pillar of the abdomen by 50%. The fall in rectus function in our patients was, however, not as predicted. Eight-six percent could still achieve a score of three. Among the controls too, the majority (67%) could only achieve a score of four. Clinically, the functional loss is, therefore, limited. Some flexion could still be performed by the contraction of the intact contralateral rectus abdominis muscle and the oblique muscles, but with increasing workload, performances (scores 4 and 5) decreased drastically.

As the recti have a complex interaction with the adjacent oblique abdominal muscles, resection of a part of the rectus abdominis muscle can cause functional changes in the oblique muscles. The “rotational curl-up” helps assess the function of the ipsilateral external oblique muscle, which laterally flexes and rotates the vertebrae. Among our TRAM flap patients, 90% had a score of two or three. Among the controls on the other hand, while only 14% achieved a score of five with both external oblique muscles, 57% had a score of four, and 29% had only a score of three. The surgery reduces the rectus function to a limited extent. The reason for this suboptimal performance is the biomechanical alteration that takes place at its insertion line. The oblique muscle does not receive sufficient counter-action from the rectus muscle after the TRAM flap has been harvested. This would cause the deflection of the insertion line laterally, thus resulting in improper function and loss of strength of the oblique muscle. This is further worsened by the limited compensation by the weak synergists for this muscle during a rotational curl-up. When comparing the external oblique function of both the operated and the nonoperated sides, there was a statistically significant difference in the scores. This contradicted the findings of Blondeel *et al*.[16] who noted that both sides had decreased function with no statistically significant difference between them. This difference in observation could be attributed to the difference in the technique adopted for the repair of the donor site. Primary suturing of the ipsilateral rectus sheath would result in increased tension and stretching which can lead to areas of permanent fibrosis and over time, to an increase in the muscle fiber length. This would cause a decreased function of the external oblique muscles on the contralateral side as well. Among our patients however, the muscluoaponeurotic deficiency was repaired with a synthetic mesh. This ensured that there was no undue tension on the contralateral side and therefore the scores obtained on “rotational curl-up” were significantly different.

However, in spite of a reduction in the strength of the upper rectus, there seems to be no proportionate decrease in the function of the lower rectus. While the control group had a mean score of 4.76, the TRAM flap patients had a mean score 4.67. Statistical analysis showed that there was no significant difference. This could be attributed to the presence of the intact synergistic muscles for the lower rectus function, namely the iliopsoas.

The questionnaire revealed that most of the patients had no subjective decrease in the strength of the abdomen (90%). No patient complained of any change in the posture or increased back pain following surgery. There existed a high degree of satisfaction among the patients with regard to the outcome of surgery (91%) while a significant number (86%) had no hesitation in recommending this reconstructive option to others who would require it. All these observations point to a successful surgical result in the patient’s perspective, and when analyzed statistically, were found to be significant. The impairment in the external oblique function seemed to contribute less to the activities of daily living. The relative impairment of abdominal wall function detected by the surgeon on clinical examination seems to contradict the good results on subjective assessment. The results of the exercise testing correlated with the high occurrence of asymmetry of the abdomen. The laxity and weakness of the abdominal musculature that caused a decreased function on objective exercise testing also caused asymmetry of the abdomen in 76% of the patients. In addition, the only patient who had a bulge in the donor site had one of the lowest scores on the straight and rotational curl-up test.

Harvesting of the medial or central part of rectus muscle was suggested by several authors in an attempt to preserve the function of the remaining muscle.[17] An increased risk to the flap vascularity was noted with this technique.[12,18] Therefore, harvesting the entire width of the rectus muscle was suggested by other authors for better flap vascularity.[12,19–22] We have also followed this principle in our TRAM flap breast reconstructions. The entire width of the anterior rectus sheath overlying the muscle in the region of the skin paddle was included. This considerably reduces the operative time. A liberal use of a synthetic mesh for repair of donor site has been advocated by many,[12,23–25] decried by some,[26] and used selectively by others.[21] While suturing the mesh to the intact contralateral anterior rectus sheath has been recommended,[25] we have sutured the mesh to the edges of the defect and intact contralateral anterior rectus sheath and the semi-lunar line. Thus, our mesh placement has both inlay and onlay elements.

The free TRAM flap based on the deep inferior epigastric system has been shown to decrease the incidence of flap vascularity-related complications in many studies.[27–29] However, a prospective multicentric study evaluating the late results of breast reconstruction with free TRAM flaps revealed abdominal wall complications occurring in up to 20% of the patients.[30] Alderman demonstrated no significant difference in abdominal wall trunk flexion between patients with free and pedicled TRAM reconstructions.[31] The two series comparing the free TRAM with the pedicled TRAM in terms of length of hospitalization, amount of pain medication used, amount of blood lost and received and complications revealed that the pedicled TRAM flap had significant economic and clinical advantages over the free TRAM flap.[28,32]

Perforator-based flaps have been suggested as an alternative to the TRAM flap in order to further limit the amount of muscle resection and thus reduce the donor site morbidity.[16,33] These flaps too have been associated with increased risk of flap loss and fat necrosis.[34,35] They are technically more challenging procedures and given the existing reimbursement environment may be difficult to justify.

Breast reconstruction conventionally involves a multispecialty team with the patient being operated upon simultaneously by the onco-surgeons and the plastic surgeons. A microvascular procedure would be technically more demanding has longer operating time and is definitely more expensive. The mean operative time for harvesting, and the inset of the TRAM flap in our series was 3 h. Doing this simultaneously with the onco-surgeons would further ensure that there is no significant prolongation of operative time.

In summary, harvesting the TRAM flap certainly results in changes to the anterior abdominal wall and these can express themselves to a variable degree in each patient. A relatively high incidence of asymmetry of the abdomen was seen. However, as it was asymptomatic in the majority and represented only a regional laxity of the abdomen wall, it does not qualify as a source of ‘morbidity.’ The total absence of hernia in any of the patients after a mean follow-up period of 15.5 months corroborates this. In most patients, synergists take over the functional movement but as the load increases (e.g., when hands are brought to the chest or neck) flexion and rotation performances decrease. However, the extent to which this impairment in function occurred clinically was less significant when the result was compared to the mediocre results of the exercise evaluation obtained from our control patients. The lack of correlation with the results of the questionnaire suggests that the impairment was functionally not significant, though statistically significant. The difficult postures and movements tested for do not form part of the activities of daily living; the patients encountered little or no difficulty in day-to-day activities. The short-operative time and the technical ease with which the breast was reconstructed are other factors in favor of this technique. Our modification of use of a wide mesh as inlay and onlay repair minimizes the donor site morbidity. It also speeds up the harvesting of flap, as one does not have to perform the dissection for preserving part of the muscle and anterior rectus sheath. This also avoids maneuvers meant for primary closure of the rectus sheath defects, which can result in distortion of umbilicus. In conclusion, the unipedicled TRAM flap should be regarded as a valuable option in breast reconstruction provided careful repair of the abdominal wall defect is undertaken using Proline mesh both as inlay and onlay techniques.
